# 1,1′-(2,5-Dimethyl­thio­phene-3,4-di­yl)diethanone

**DOI:** 10.1107/S1600536811029710

**Published:** 2011-08-02

**Authors:** Chengpeng Li, Qiaozheng Qi, Sheng Wang, Guohua Ding

**Affiliations:** a Department of Chemistry and Bioengineering, Guilin University of Technology, Guilin 541004, People’s Republic of China; b School of Chemistry Science and Technology, Zhanjiang Normal University, Development Center for New Materials Engineering and Technology in Universities of Guangdong, Zhanjiang 524048, People’s Republic of China

## Abstract

The title compound, C_10_H_12_O_2_S, crystallizes with four mol­ecules in the asymmetric unit. The main conformational difference between these mol­ecules is the orientation of the acetyl groups with respect to the ring. Whereas one acetyl group is only slightly twisted with respect to the thio­phene ring [C—C—C—O torsion angles = 165.7 (4), −164.6 (4), 164.3 (4) and −163.6 (4)°], the other acetyl group is markly twisted out of the ring plane [C—C—C—O torsion angles = −61.2 (6), 61.3 (7), −59.7 (7) and 59.9 (6)°]. In the crystal, mol­ecules are linked by weak C—H⋯O inter­actions into infinite chains along the *c* axis.

## Related literature

For the synthesis of the title compound, see: Li *et al.* (2011 ▶); Wang *et al.* (2004 ▶). For a related structure, see: Yu *et al.* (2010 ▶).
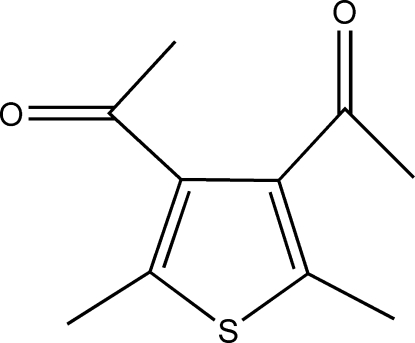


## Experimental

### 

#### Crystal data

C_10_H_12_O_2_S
*M* *_r_* = 196.26
Monoclinic, 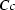

*a* = 12.142 (2) Å
*b* = 12.129 (2) Å
*c* = 27.446 (6) Å
β = 99.387 (2)°
*V* = 3987.8 (14) Å^3^
*Z* = 16
Mo *K*α radiation
μ = 0.29 mm^−1^
*T* = 296 K
0.38 × 0.30 × 0.21 mm

#### Data collection

Bruker SMART APEXII CCD area-detector diffractometer
Absorption correction: multi-scan (*SADABS*; Sheldrick, 2004 ▶) *T*_min_ = 0.898, *T*_max_ = 0.942
14665 measured reflections
7205 independent reflections
4969 reflections with *I* > 2σ(*I*)
*R*_int_ = 0.031

#### Refinement

*R*[*F* ^2^ > 2σ(*F* ^2^)] = 0.043
*wR*(*F* ^2^) = 0.092
*S* = 1.02
7205 reflections
485 parameters
2 restraints
H-atom parameters constrained
Δρ_max_ = 0.17 e Å^−3^
Δρ_min_ = −0.23 e Å^−3^
Absolute structure: Flack (1983 ▶), 3486 Friedel pairs
Flack parameter: 0.05 (7)

### 

Data collection: *APEX2* (Bruker, 2004 ▶); cell refinement: *SAINT-Plus* (Bruker, 2001 ▶); data reduction: *SAINT-Plus*; program(s) used to solve structure: *SHELXS97* (Sheldrick, 2008 ▶); program(s) used to refine structure: *SHELXL97* (Sheldrick, 2008 ▶); molecular graphics: *SHELXTL* (Sheldrick, 2008 ▶); software used to prepare material for publication: *SHELXL97*.

## Supplementary Material

Crystal structure: contains datablock(s) I, global. DOI: 10.1107/S1600536811029710/bt5559sup1.cif

Structure factors: contains datablock(s) I. DOI: 10.1107/S1600536811029710/bt5559Isup2.hkl

Supplementary material file. DOI: 10.1107/S1600536811029710/bt5559Isup3.cml

Additional supplementary materials:  crystallographic information; 3D view; checkCIF report

## Figures and Tables

**Table 1:** Hydrogen-bond geometry (Å, °)

*D*—H⋯*A*	*D*—H	H⋯*A*	*D*⋯*A*	*D*—H⋯*A*
C35—H35*C*⋯O6^i^	0.96	2.44	3.276 (6)	145
C39—H39*A*⋯O6^i^	0.96	2.56	3.435 (6)	152

Symmetry code: (i) 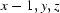.

**Fig. 1.**
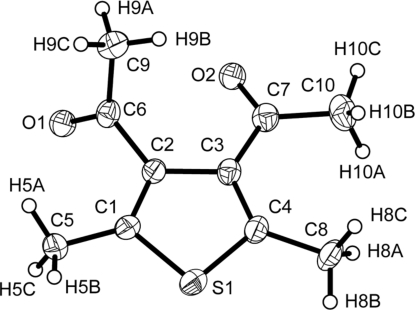
Molecular structure of one molecule in the asymmetric unit of the title compound, showing 30% probability displacement ellipsoids.

**Fig. 2.**
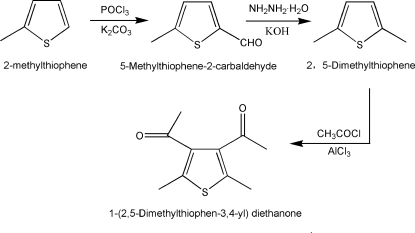
The synthetic route to the title compound.

## supplementary crystallographic information

### Comment

Azomethines are an important class of compounds which have been intensively investigated owning to their strong coordination capability, antibacterial activity, antitumor property and so on. Considering   this, on our way to getting novel photochromic molecules on which we focused in the past few years, we first designed and synthesized a key intermediate 1-(2,5-Dimethylthiophen-3,4-yl) diethanone, which has two carbonyl groups. Usually, azomethines are obtained by the condensation of carbonyl compounds with primary amines. Herein, the design and synthesis of this compound provides a wide space for the new azomethines of thiophene. Recently the introduction of Schiff base ligands into photochromic diarylethene system and their photochromic properties in solution has been reported (Li *et al.*, 2011). We are trying to push forward that work through introducing the title compound to the system. Moreover, in our recent study we also found that the title compound played a good role in the synthesis of Schiff-base macrocycles. When we took different type or the length of chain diamines, we got varying size of the macrocycles and some of them had good ability of cooperation with metals.

### Experimental

We used 2-methylthiophene as the starting material *via*, in turn, Vilsmeier, Wolff-Kishner-Huang, and Friedel-Crafts reactions and got the title compound. The synthetic processes are as follows:

5-Methylthiophene-2-carbaldehyde

To a 10 g anhydrous dimethylformamide solution of 2-methylthiophene (10 g, 0.1 mol), a (17 g, 0.11 mol) phosphorus oxychloride (POCl_3_) was added drop by drop slowly at 0°C. After addition, the ice bath was removed and the mixture was stirred for 0.5 h at room temperature. Then, the reddish solution was heated slowly to reflux. After refluxed for 1 h and cooled to room temperature, the mixture was poured into ice water and K_2_CO_3_ was added until pH=10. The mixture was extracted with diethyl ether (3×25 ml). The combined organic layers were washed with a saturated NaCl solution (2×25 ml) and H_2_O (1×25 ml), dried (MgSO_4_), filtered and the solvents evaporated in vacuum to yield: 10.8 g, 84.2%.

2,5-Dimethylthiophene

To a 360 ml e thyl glycol solution of 5-methyl-thiophene-2-carbaldehyde (112 g, 0.9 mol), a 120 ml hydrazine hydrate (85%) was added in 1000 ml flask. The mixture was refluxed for 0.5 h, and then evaporated the excessive water and hydrazine hydrate until the oil drops showed up. After the evaporation, KOH (20 g, 0.3 mol) was added in portions to the cooled mixture. Then refluxed for 0.5 h, distilled and the mixture of oil and water was washed with a saturated NaCl solution (3× 25 ml), After being extracted, the organic phase was distilled and the fraction boiling between 134 °C and 135 °C was collected to yield 110 g, 91%.

1-(2,5-Dimethylthiophen-3,4-yl) diethanone

To a 200 ml dichloromethane solution of anhydrous aluminium chloride (41 g,0.3 mol), a 11 ml dichloromethane solution of acetyl chloride(16.4 g, 0.21 mol) and 15 ml dichloromethane solution of 2,5-Dimethylthiophene (23.5 g, 0.21 mol) was added dropwise in turn at 0°C. After addition, the reaction mixture was stirred for 8 h at room temperature. Then the mixture was poured into 45 ml ice-hydrochloric acid. The product was extracted with dichloromethane and the solution was dried (MgSO_4_). After evaporation of the solvent, the pure product was obtained as a yellow solid (33.5 g, 81%) by column chromatography with petroleum/ethyl acetate(8:1) as eluent.

### Refinement

H atoms were geometrically positioned with C-H = 0.96Å and U(H)=1.5U_eq_(C).

### Crystal data

**Table:**

C_10_H_12_O_2_S	*D*_x_ = 1.308 Mg m^−^^3^
*M**_r_* = 196.26	Melting point: 363 K
Monoclinic, *C**c*	Mo *K*α radiation, λ = 0.71073 Å
*a* = 12.142 (2) Å	Cell parameters from 3384 reflections
*b* = 12.129 (2) Å	θ = 2.4–23.9°
*c* = 27.446 (6) Å	µ = 0.29 mm^−^^1^
β = 99.387 (2)°	*T* = 296 K
*V* = 3987.8 (14) Å^3^	Block, colourless
*Z* = 16	0.38 × 0.30 × 0.21 mm
*F*(000) = 1664	

### Data collection

**Table:**

Bruker SMART APEXII CCD area-detector diffractometer	7205 independent reflections
Radiation source: fine-focus sealed tube	4969 reflections with *I* > 2σ(*I*)
graphite	*R*_int_ = 0.031
phi and ω scans	θ_max_ = 25.5°, θ_min_ = 2.4°
Absorption correction: multi-scan (*SADABS*; Sheldrick, 2004)	*h* = −14→14
*T*_min_ = 0.898, *T*_max_ = 0.942	*k* = −14→14
14665 measured reflections	*l* = −32→33

### Refinement

**Table:**

Refinement on *F*^2^	Secondary atom site location: difference Fourier map
Least-squares matrix: full	Hydrogen site location: inferred from neighbouring sites
*R*[*F*^2^ > 2σ(*F*^2^)] = 0.043	H-atom parameters constrained
*wR*(*F*^2^) = 0.092	*w* = 1/[σ^2^(*F*_o_^2^) + (0.0329*P*)^2^ + 1.3235*P*] where *P* = (*F*_o_^2^ + 2*F*_c_^2^)/3
*S* = 1.02	(Δ/σ)_max_ = 0.001
7205 reflections	Δρ_max_ = 0.17 e Å^−^^3^
485 parameters	Δρ_min_ = −0.23 e Å^−^^3^
2 restraints	Absolute structure: Flack (1983), 3486 Friedel pairs
Primary atom site location: structure-invariant direct methods	Flack parameter: 0.05 (7)

### Special details

**Table:**

Geometry. All e.s.d.'s (except the e.s.d. in the dihedral angle between two l.s. planes) are estimated using the full covariance matrix. The cell e.s.d.'s are taken into account individually in the estimation of e.s.d.'s in distances, angles and torsion angles; correlations between e.s.d.'s in cell parameters are only used when they are defined by crystal symmetry. An approximate (isotropic) treatment of cell e.s.d.'s is used for estimating e.s.d.'s involving l.s. planes.
Refinement. Refinement of *F*^2^ against ALL reflections. The weighted *R*-factor *wR* and goodness of fit *S* are based on *F*^2^, conventional *R*-factors *R* are based on *F*, with *F* set to zero for negative *F*^2^. The threshold expression of *F*^2^ > σ(*F*^2^) is used only for calculating *R*-factors(gt) *etc*. and is not relevant to the choice of reflections for refinement. *R*-factors based on *F*^2^ are statistically about twice as large as those based on *F*, and *R*- factors based on ALL data will be even larger.

### Fractional atomic coordinates and isotropic or equivalent isotropic displacement parameters (Å^2^)

**Table:**

	*x*	*y*	*z*	*U*_iso_*/*U*_eq_	
C1	0.1299 (4)	0.1176 (4)	0.4644 (2)	0.0439 (13)	
C2	0.0669 (4)	0.1446 (4)	0.4207 (2)	0.0401 (13)	
C3	−0.0256 (4)	0.0686 (3)	0.40549 (18)	0.0402 (12)	
C4	−0.0279 (3)	−0.0142 (3)	0.43893 (16)	0.0451 (10)	
C5	0.2301 (5)	0.1710 (4)	0.4926 (2)	0.0554 (16)	
H5A	0.2746	0.2015	0.4700	0.083*	
H5B	0.2731	0.1173	0.5132	0.083*	
H5C	0.2076	0.2287	0.5128	0.083*	
C6	0.0987 (4)	0.2391 (4)	0.3911 (2)	0.0437 (13)	
C7	−0.1085 (4)	0.0924 (4)	0.36101 (17)	0.0473 (10)	
C8	−0.1080 (4)	−0.1097 (3)	0.44085 (17)	0.0620 (13)	
H8A	−0.1834	−0.0828	0.4352	0.093*	
H8B	−0.0927	−0.1441	0.4727	0.093*	
H8C	−0.0985	−0.1626	0.4158	0.093*	
C9	0.1385 (4)	0.2160 (4)	0.34303 (15)	0.0575 (12)	
H9A	0.1178	0.2760	0.3206	0.086*	
H9B	0.1049	0.1491	0.3290	0.086*	
H9C	0.2182	0.2081	0.3488	0.086*	
C10	−0.1952 (4)	0.0083 (4)	0.34150 (19)	0.0743 (15)	
H10A	−0.2475	0.0008	0.3640	0.112*	
H10B	−0.1598	−0.0614	0.3381	0.112*	
H10C	−0.2339	0.0316	0.3099	0.112*	
C11	0.2336 (3)	0.7569 (3)	0.43718 (15)	0.0421 (10)	
C12	0.1384 (4)	0.7388 (4)	0.40351 (18)	0.0393 (12)	
C13	0.0706 (4)	0.6523 (4)	0.4191 (2)	0.0379 (13)	
C14	0.1126 (4)	0.6077 (4)	0.4636 (2)	0.0433 (13)	
C15	0.3259 (4)	0.8407 (4)	0.43894 (17)	0.0675 (13)	
H15A	0.3723	0.8218	0.4150	0.101*	
H15B	0.3700	0.8411	0.4713	0.101*	
H15C	0.2941	0.9124	0.4316	0.101*	
C16	0.0998 (4)	0.8043 (3)	0.35802 (16)	0.0460 (10)	
C17	−0.0341 (4)	0.6062 (4)	0.38910 (18)	0.0432 (12)	
C18	0.0672 (5)	0.5167 (4)	0.4928 (2)	0.0597 (17)	
H18A	0.0370	0.4588	0.4707	0.090*	
H18B	0.0096	0.5460	0.5092	0.090*	
H18C	0.1264	0.4877	0.5169	0.090*	
C19	0.1763 (4)	0.8807 (4)	0.33769 (18)	0.0662 (13)	
H19A	0.1419	0.9050	0.3055	0.099*	
H19B	0.2448	0.8433	0.3353	0.099*	
H19C	0.1915	0.9433	0.3591	0.099*	
C20	−0.0254 (4)	0.5480 (4)	0.34209 (16)	0.0579 (12)	
H20A	−0.0164	0.4704	0.3483	0.087*	
H20B	0.0378	0.5756	0.3290	0.087*	
H20C	−0.0922	0.5605	0.3187	0.087*	
C21	0.3487 (3)	−0.0066 (3)	0.13375 (16)	0.0452 (10)	
C22	0.4442 (4)	0.0093 (4)	0.16737 (18)	0.0397 (12)	
C23	0.5136 (4)	0.0960 (4)	0.1516 (2)	0.0395 (14)	
C24	0.4702 (4)	0.1402 (4)	0.1073 (2)	0.0412 (13)	
C25	0.2538 (4)	−0.0864 (4)	0.13179 (18)	0.0595 (12)	
H25A	0.2771	−0.1578	0.1223	0.089*	
H25B	0.1918	−0.0614	0.1081	0.089*	
H25C	0.2317	−0.0911	0.1638	0.089*	
C26	0.4849 (4)	−0.0570 (3)	0.21115 (17)	0.0468 (10)	
C27	0.6200 (4)	0.1393 (4)	0.18038 (19)	0.0457 (13)	
C28	0.5153 (5)	0.2308 (5)	0.0799 (2)	0.0621 (17)	
H28A	0.4779	0.2985	0.0851	0.093*	
H28B	0.5034	0.2137	0.0452	0.093*	
H28C	0.5939	0.2387	0.0915	0.093*	
C29	0.4078 (4)	−0.1369 (4)	0.23122 (17)	0.0653 (13)	
H29A	0.3862	−0.1943	0.2075	0.098*	
H29B	0.3425	−0.0985	0.2375	0.098*	
H29C	0.4457	−0.1687	0.2614	0.098*	
C30	0.6137 (4)	0.1957 (3)	0.22774 (17)	0.0581 (12)	
H30A	0.6874	0.2024	0.2465	0.087*	
H30B	0.5677	0.1535	0.2462	0.087*	
H30C	0.5820	0.2677	0.2212	0.087*	
C31	−0.0373 (4)	0.1242 (4)	0.1086 (2)	0.0409 (13)	
C32	0.0215 (4)	0.0999 (4)	0.1535 (2)	0.0373 (13)	
C33	0.1135 (4)	0.1744 (3)	0.16831 (18)	0.0390 (12)	
C34	0.1199 (3)	0.2560 (3)	0.13392 (15)	0.0438 (10)	
C35	−0.1391 (5)	0.0656 (4)	0.0813 (2)	0.0583 (17)	
H35A	−0.1332	−0.0119	0.0883	0.087*	
H35B	−0.1435	0.0773	0.0464	0.087*	
H35C	−0.2050	0.0943	0.0919	0.087*	
C36	−0.0130 (4)	0.0059 (4)	0.1833 (2)	0.0445 (13)	
C37	0.1960 (3)	0.1528 (4)	0.21315 (17)	0.0464 (10)	
C38	0.1999 (4)	0.3480 (3)	0.13055 (18)	0.0620 (13)	
H38A	0.2176	0.3837	0.1621	0.093*	
H38B	0.1667	0.4004	0.1063	0.093*	
H38C	0.2670	0.3191	0.1211	0.093*	
C39	−0.0535 (4)	0.0325 (4)	0.22985 (16)	0.0580 (12)	
H39A	−0.1235	0.0707	0.2225	0.087*	
H39B	0.0001	0.0785	0.2500	0.087*	
H39C	−0.0634	−0.0345	0.2473	0.087*	
C40	0.2819 (3)	0.2373 (4)	0.23302 (17)	0.0615 (13)	
H40A	0.3211	0.2133	0.2645	0.092*	
H40B	0.2458	0.3064	0.2369	0.092*	
H40C	0.3339	0.2461	0.2104	0.092*	
O1	0.1038 (3)	0.3320 (2)	0.40784 (13)	0.0705 (9)	
O2	−0.1062 (2)	0.1817 (3)	0.34011 (12)	0.0668 (9)	
O3	0.0032 (3)	0.7933 (3)	0.33761 (11)	0.0626 (9)	
O4	−0.1202 (2)	0.6078 (3)	0.40579 (13)	0.0688 (9)	
O5	0.5817 (3)	−0.0474 (2)	0.23140 (12)	0.0665 (9)	
O6	0.7055 (2)	0.1377 (3)	0.16305 (12)	0.0677 (9)	
O7	−0.0166 (3)	−0.0868 (2)	0.16659 (12)	0.0658 (8)	
O8	0.1926 (2)	0.0637 (2)	0.23397 (12)	0.0605 (8)	
S1	0.07809 (11)	−0.00057 (11)	0.48785 (5)	0.0531 (4)	
S2	0.23678 (11)	0.67089 (12)	0.48713 (5)	0.0541 (4)	
S3	0.34281 (10)	0.08175 (12)	0.08483 (5)	0.0522 (4)	
S4	0.01417 (10)	0.23984 (11)	0.08442 (5)	0.0525 (4)	

### Atomic displacement parameters (Å^2^)

**Table:**

	*U*^11^	*U*^22^	*U*^33^	*U*^12^	*U*^13^	*U*^23^
C1	0.043 (3)	0.049 (3)	0.041 (3)	−0.005 (2)	0.011 (2)	0.004 (3)
C2	0.041 (3)	0.040 (3)	0.041 (4)	−0.005 (3)	0.010 (3)	−0.001 (3)
C3	0.036 (2)	0.040 (3)	0.046 (3)	−0.002 (2)	0.009 (2)	−0.003 (2)
C4	0.046 (2)	0.040 (2)	0.052 (3)	−0.0078 (19)	0.015 (2)	−0.003 (2)
C5	0.049 (3)	0.073 (3)	0.043 (4)	−0.018 (3)	0.006 (3)	−0.005 (3)
C6	0.034 (3)	0.043 (3)	0.053 (3)	−0.002 (2)	0.005 (2)	0.003 (2)
C7	0.039 (2)	0.051 (3)	0.053 (3)	0.001 (2)	0.010 (2)	−0.002 (2)
C8	0.066 (3)	0.053 (3)	0.071 (3)	−0.023 (2)	0.025 (3)	0.003 (2)
C9	0.057 (3)	0.062 (3)	0.056 (3)	0.002 (2)	0.015 (2)	0.009 (2)
C10	0.050 (3)	0.087 (4)	0.078 (4)	−0.015 (3)	−0.012 (3)	0.000 (3)
C11	0.039 (2)	0.045 (2)	0.044 (2)	−0.0095 (19)	0.0109 (19)	−0.005 (2)
C12	0.040 (3)	0.036 (2)	0.042 (3)	−0.003 (2)	0.008 (2)	−0.0013 (19)
C13	0.040 (3)	0.034 (3)	0.039 (3)	−0.005 (2)	0.005 (3)	−0.003 (2)
C14	0.044 (3)	0.043 (3)	0.042 (3)	−0.007 (2)	0.005 (3)	−0.003 (2)
C15	0.057 (3)	0.073 (3)	0.069 (3)	−0.030 (3)	0.002 (2)	−0.003 (3)
C16	0.051 (3)	0.041 (3)	0.046 (3)	0.009 (2)	0.009 (2)	0.006 (2)
C17	0.043 (3)	0.039 (3)	0.047 (3)	0.001 (2)	0.003 (2)	0.006 (2)
C18	0.070 (4)	0.061 (3)	0.049 (4)	−0.018 (3)	0.012 (3)	0.001 (3)
C19	0.069 (3)	0.057 (3)	0.072 (3)	−0.015 (2)	0.010 (3)	0.016 (3)
C20	0.059 (3)	0.058 (3)	0.052 (3)	−0.002 (2)	−0.004 (2)	−0.011 (2)
C21	0.041 (2)	0.043 (2)	0.053 (3)	−0.008 (2)	0.010 (2)	−0.007 (2)
C22	0.033 (3)	0.041 (3)	0.046 (3)	0.000 (2)	0.011 (2)	−0.003 (2)
C23	0.032 (3)	0.040 (3)	0.047 (4)	0.001 (2)	0.009 (3)	−0.006 (3)
C24	0.039 (3)	0.045 (3)	0.040 (3)	−0.008 (2)	0.008 (2)	−0.009 (3)
C25	0.048 (2)	0.061 (3)	0.072 (3)	−0.024 (2)	0.016 (2)	−0.004 (3)
C26	0.049 (3)	0.041 (3)	0.051 (3)	−0.004 (2)	0.011 (2)	−0.007 (2)
C27	0.039 (3)	0.042 (3)	0.055 (3)	−0.005 (2)	0.005 (2)	0.002 (2)
C28	0.070 (4)	0.064 (3)	0.050 (4)	−0.016 (3)	0.004 (3)	0.005 (3)
C29	0.076 (3)	0.052 (3)	0.067 (3)	−0.005 (2)	0.010 (3)	0.020 (2)
C30	0.060 (3)	0.054 (3)	0.056 (3)	−0.006 (2)	−0.002 (2)	−0.006 (2)
C31	0.046 (3)	0.039 (3)	0.039 (3)	−0.006 (2)	0.011 (3)	−0.002 (2)
C32	0.034 (3)	0.037 (3)	0.042 (4)	−0.002 (2)	0.010 (3)	0.000 (2)
C33	0.043 (3)	0.034 (2)	0.041 (3)	−0.002 (2)	0.010 (2)	−0.003 (2)
C34	0.046 (2)	0.041 (2)	0.047 (3)	−0.0033 (19)	0.012 (2)	0.001 (2)
C35	0.064 (4)	0.059 (3)	0.050 (4)	−0.013 (3)	0.002 (3)	0.006 (3)
C36	0.040 (3)	0.043 (3)	0.050 (3)	−0.003 (2)	0.004 (2)	0.006 (2)
C37	0.037 (2)	0.052 (3)	0.050 (3)	0.002 (2)	0.008 (2)	−0.003 (2)
C38	0.062 (3)	0.049 (3)	0.075 (3)	−0.021 (2)	0.012 (3)	0.000 (2)
C39	0.053 (3)	0.065 (3)	0.057 (3)	−0.004 (2)	0.013 (2)	0.014 (2)
C40	0.046 (3)	0.066 (3)	0.070 (4)	−0.011 (2)	0.000 (2)	0.001 (3)
O1	0.089 (2)	0.0401 (18)	0.084 (3)	−0.0120 (17)	0.0214 (19)	−0.0049 (17)
O2	0.061 (2)	0.063 (2)	0.071 (2)	0.0018 (17)	−0.0037 (17)	0.0143 (18)
O3	0.0558 (19)	0.067 (2)	0.060 (2)	−0.0011 (15)	−0.0072 (16)	0.0114 (16)
O4	0.0389 (18)	0.089 (2)	0.080 (2)	−0.0106 (16)	0.0149 (17)	−0.0050 (19)
O5	0.0523 (19)	0.066 (2)	0.077 (2)	0.0014 (16)	0.0002 (18)	0.0154 (18)
O6	0.0384 (18)	0.092 (2)	0.073 (2)	−0.0116 (17)	0.0114 (16)	−0.0069 (19)
O7	0.085 (2)	0.0436 (18)	0.071 (2)	−0.0106 (17)	0.0194 (17)	−0.0003 (16)
O8	0.0559 (19)	0.0557 (19)	0.066 (2)	−0.0016 (15)	−0.0001 (15)	0.0159 (16)
S1	0.0551 (9)	0.0545 (8)	0.0502 (9)	−0.0083 (6)	0.0100 (7)	0.0104 (6)
S2	0.0500 (8)	0.0611 (8)	0.0471 (9)	−0.0126 (6)	−0.0039 (6)	0.0002 (7)
S3	0.0482 (8)	0.0592 (8)	0.0464 (9)	−0.0120 (6)	−0.0008 (6)	−0.0015 (7)
S4	0.0577 (9)	0.0525 (8)	0.0468 (9)	−0.0106 (7)	0.0074 (7)	0.0097 (7)

### Geometric parameters (Å, °)

**Table:**

C1—C2	1.352 (7)	C21—C22	1.373 (6)
C1—C5	1.480 (7)	C21—C25	1.499 (5)
C1—S1	1.731 (5)	C21—S3	1.710 (4)
C2—C3	1.461 (7)	C22—C23	1.456 (6)
C2—C6	1.491 (7)	C22—C26	1.463 (6)
C3—C4	1.364 (6)	C23—C24	1.355 (7)
C3—C7	1.478 (6)	C23—C27	1.495 (7)
C4—C8	1.519 (5)	C24—C28	1.487 (7)
C4—S1	1.710 (5)	C24—S3	1.721 (5)
C5—H5A	0.9600	C25—H25A	0.9600
C5—H5B	0.9600	C25—H25B	0.9600
C5—H5C	0.9600	C25—H25C	0.9600
C6—O1	1.215 (5)	C26—O5	1.221 (5)
C6—C9	1.504 (6)	C26—C29	1.513 (6)
C7—O2	1.228 (5)	C27—O6	1.211 (5)
C7—C10	1.501 (6)	C27—C30	1.481 (6)
C8—H8A	0.9600	C28—H28A	0.9600
C8—H8B	0.9600	C28—H28B	0.9600
C8—H8C	0.9600	C28—H28C	0.9600
C9—H9A	0.9600	C29—H29A	0.9600
C9—H9B	0.9600	C29—H29B	0.9600
C9—H9C	0.9600	C29—H29C	0.9600
C10—H10A	0.9600	C30—H30A	0.9600
C10—H10B	0.9600	C30—H30B	0.9600
C10—H10C	0.9600	C30—H30C	0.9600
C11—C12	1.374 (6)	C31—C32	1.353 (7)
C11—C15	1.508 (5)	C31—C35	1.514 (7)
C11—S2	1.718 (4)	C31—S4	1.713 (5)
C12—C13	1.441 (6)	C32—C33	1.443 (7)
C12—C16	1.490 (6)	C32—C36	1.502 (7)
C13—C14	1.358 (7)	C33—C34	1.379 (5)
C13—C17	1.505 (7)	C33—C37	1.478 (6)
C14—C18	1.519 (7)	C34—C38	1.492 (5)
C14—S2	1.720 (5)	C34—S4	1.722 (4)
C15—H15A	0.9600	C35—H35A	0.9600
C15—H15B	0.9600	C35—H35B	0.9600
C15—H15C	0.9600	C35—H35C	0.9600
C16—O3	1.221 (5)	C36—O7	1.213 (5)
C16—C19	1.484 (6)	C36—C39	1.477 (7)
C17—O4	1.209 (5)	C37—O8	1.226 (5)
C17—C20	1.489 (6)	C37—C40	1.500 (6)
C18—H18A	0.9600	C38—H38A	0.9600
C18—H18B	0.9600	C38—H38B	0.9600
C18—H18C	0.9600	C38—H38C	0.9600
C19—H19A	0.9600	C39—H39A	0.9600
C19—H19B	0.9600	C39—H39B	0.9600
C19—H19C	0.9600	C39—H39C	0.9600
C20—H20A	0.9600	C40—H40A	0.9600
C20—H20B	0.9600	C40—H40B	0.9600
C20—H20C	0.9600	C40—H40C	0.9600
			
C2—C1—C5	130.9 (5)	C25—C21—S3	116.3 (3)
C2—C1—S1	110.1 (4)	C21—C22—C23	111.6 (4)
C5—C1—S1	119.0 (4)	C21—C22—C26	127.6 (4)
C1—C2—C3	113.5 (4)	C23—C22—C26	120.4 (4)
C1—C2—C6	120.7 (5)	C24—C23—C22	113.2 (5)
C3—C2—C6	125.7 (5)	C24—C23—C27	120.7 (4)
C4—C3—C2	111.8 (4)	C22—C23—C27	126.1 (5)
C4—C3—C7	127.8 (4)	C23—C24—C28	128.7 (5)
C2—C3—C7	120.1 (4)	C23—C24—S3	110.7 (4)
C3—C4—C8	132.0 (4)	C28—C24—S3	120.5 (4)
C3—C4—S1	111.2 (3)	C21—C25—H25A	109.5
C8—C4—S1	116.8 (3)	C21—C25—H25B	109.5
C1—C5—H5A	109.5	H25A—C25—H25B	109.5
C1—C5—H5B	109.5	C21—C25—H25C	109.5
H5A—C5—H5B	109.5	H25A—C25—H25C	109.5
C1—C5—H5C	109.5	H25B—C25—H25C	109.5
H5A—C5—H5C	109.5	O5—C26—C22	119.5 (4)
H5B—C5—H5C	109.5	O5—C26—C29	120.1 (4)
O1—C6—C2	120.6 (5)	C22—C26—C29	120.3 (4)
O1—C6—C9	120.0 (5)	O6—C27—C30	121.5 (4)
C2—C6—C9	118.9 (4)	O6—C27—C23	120.6 (5)
O2—C7—C3	119.5 (4)	C30—C27—C23	117.5 (4)
O2—C7—C10	120.1 (4)	C24—C28—H28A	109.5
C3—C7—C10	120.4 (4)	C24—C28—H28B	109.5
C4—C8—H8A	109.5	H28A—C28—H28B	109.5
C4—C8—H8B	109.5	C24—C28—H28C	109.5
H8A—C8—H8B	109.5	H28A—C28—H28C	109.5
C4—C8—H8C	109.5	H28B—C28—H28C	109.5
H8A—C8—H8C	109.5	C26—C29—H29A	109.5
H8B—C8—H8C	109.5	C26—C29—H29B	109.5
C6—C9—H9A	109.5	H29A—C29—H29B	109.5
C6—C9—H9B	109.5	C26—C29—H29C	109.5
H9A—C9—H9B	109.5	H29A—C29—H29C	109.5
C6—C9—H9C	109.5	H29B—C29—H29C	109.5
H9A—C9—H9C	109.5	C27—C30—H30A	109.5
H9B—C9—H9C	109.5	C27—C30—H30B	109.5
C7—C10—H10A	109.5	H30A—C30—H30B	109.5
C7—C10—H10B	109.5	C27—C30—H30C	109.5
H10A—C10—H10B	109.5	H30A—C30—H30C	109.5
C7—C10—H10C	109.5	H30B—C30—H30C	109.5
H10A—C10—H10C	109.5	C32—C31—C35	128.0 (5)
H10B—C10—H10C	109.5	C32—C31—S4	111.1 (4)
C12—C11—C15	131.9 (4)	C35—C31—S4	120.8 (4)
C12—C11—S2	110.3 (3)	C31—C32—C33	112.9 (4)
C15—C11—S2	117.6 (3)	C31—C32—C36	120.7 (5)
C11—C12—C13	112.0 (4)	C33—C32—C36	126.4 (5)
C11—C12—C16	126.4 (4)	C34—C33—C32	112.6 (4)
C13—C12—C16	121.3 (4)	C34—C33—C37	126.2 (4)
C14—C13—C12	114.1 (5)	C32—C33—C37	120.8 (4)
C14—C13—C17	120.3 (4)	C33—C34—C38	133.6 (4)
C12—C13—C17	125.5 (5)	C33—C34—S4	109.9 (3)
C13—C14—C18	130.4 (5)	C38—C34—S4	116.4 (3)
C13—C14—S2	109.8 (4)	C31—C35—H35A	109.5
C18—C14—S2	119.8 (4)	C31—C35—H35B	109.5
C11—C15—H15A	109.5	H35A—C35—H35B	109.5
C11—C15—H15B	109.5	C31—C35—H35C	109.5
H15A—C15—H15B	109.5	H35A—C35—H35C	109.5
C11—C15—H15C	109.5	H35B—C35—H35C	109.5
H15A—C15—H15C	109.5	O7—C36—C39	122.2 (4)
H15B—C15—H15C	109.5	O7—C36—C32	119.5 (5)
O3—C16—C19	120.6 (4)	C39—C36—C32	117.9 (4)
O3—C16—C12	118.3 (4)	O8—C37—C33	118.3 (4)
C19—C16—C12	121.2 (4)	O8—C37—C40	120.5 (4)
O4—C17—C20	121.6 (4)	C33—C37—C40	121.2 (4)
O4—C17—C13	119.5 (5)	C34—C38—H38A	109.5
C20—C17—C13	118.5 (4)	C34—C38—H38B	109.5
C14—C18—H18A	109.5	H38A—C38—H38B	109.5
C14—C18—H18B	109.5	C34—C38—H38C	109.5
H18A—C18—H18B	109.5	H38A—C38—H38C	109.5
C14—C18—H18C	109.5	H38B—C38—H38C	109.5
H18A—C18—H18C	109.5	C36—C39—H39A	109.5
H18B—C18—H18C	109.5	C36—C39—H39B	109.5
C16—C19—H19A	109.5	H39A—C39—H39B	109.5
C16—C19—H19B	109.5	C36—C39—H39C	109.5
H19A—C19—H19B	109.5	H39A—C39—H39C	109.5
C16—C19—H19C	109.5	H39B—C39—H39C	109.5
H19A—C19—H19C	109.5	C37—C40—H40A	109.5
H19B—C19—H19C	109.5	C37—C40—H40B	109.5
C17—C20—H20A	109.5	H40A—C40—H40B	109.5
C17—C20—H20B	109.5	C37—C40—H40C	109.5
H20A—C20—H20B	109.5	H40A—C40—H40C	109.5
C17—C20—H20C	109.5	H40B—C40—H40C	109.5
H20A—C20—H20C	109.5	C4—S1—C1	93.5 (2)
H20B—C20—H20C	109.5	C11—S2—C14	93.8 (2)
C22—C21—C25	132.4 (4)	C21—S3—C24	93.3 (2)
C22—C21—S3	111.2 (3)	C31—S4—C34	93.4 (2)
			
C5—C1—C2—C3	179.5 (5)	C22—C23—C24—C28	178.8 (5)
S1—C1—C2—C3	0.0 (5)	C27—C23—C24—C28	1.3 (8)
C5—C1—C2—C6	3.7 (9)	C22—C23—C24—S3	2.1 (5)
S1—C1—C2—C6	−175.8 (4)	C27—C23—C24—S3	−175.4 (4)
C1—C2—C3—C4	−0.3 (6)	C21—C22—C26—O5	164.3 (4)
C6—C2—C3—C4	175.2 (4)	C23—C22—C26—O5	−7.8 (6)
C1—C2—C3—C7	174.2 (4)	C21—C22—C26—C29	−15.6 (7)
C6—C2—C3—C7	−10.2 (7)	C23—C22—C26—C29	172.2 (4)
C2—C3—C4—C8	177.8 (4)	C24—C23—C27—O6	−59.7 (7)
C7—C3—C4—C8	3.8 (8)	C22—C23—C27—O6	123.1 (5)
C2—C3—C4—S1	0.4 (5)	C24—C23—C27—C30	113.2 (5)
C7—C3—C4—S1	−173.6 (4)	C22—C23—C27—C30	−64.0 (6)
C1—C2—C6—O1	−61.2 (7)	C35—C31—C32—C33	−179.4 (5)
C3—C2—C6—O1	123.5 (5)	S4—C31—C32—C33	−2.1 (5)
C1—C2—C6—C9	111.0 (5)	C35—C31—C32—C36	−1.3 (8)
C3—C2—C6—C9	−64.2 (6)	S4—C31—C32—C36	176.1 (4)
C4—C3—C7—O2	165.7 (4)	C31—C32—C33—C34	1.5 (6)
C2—C3—C7—O2	−7.9 (7)	C36—C32—C33—C34	−176.6 (4)
C4—C3—C7—C10	−14.1 (7)	C31—C32—C33—C37	−172.0 (4)
C2—C3—C7—C10	172.3 (4)	C36—C32—C33—C37	9.9 (7)
C15—C11—C12—C13	−176.3 (4)	C32—C33—C34—C38	−177.1 (4)
S2—C11—C12—C13	−1.2 (5)	C37—C33—C34—C38	−4.0 (7)
C15—C11—C12—C16	−1.7 (8)	C32—C33—C34—S4	−0.2 (5)
S2—C11—C12—C16	173.4 (4)	C37—C33—C34—S4	172.9 (4)
C11—C12—C13—C14	0.9 (6)	C31—C32—C36—O7	59.9 (6)
C16—C12—C13—C14	−174.0 (4)	C33—C32—C36—O7	−122.2 (5)
C11—C12—C13—C17	−174.9 (4)	C31—C32—C36—C39	−113.3 (5)
C16—C12—C13—C17	10.1 (7)	C33—C32—C36—C39	64.6 (6)
C12—C13—C14—C18	179.7 (5)	C34—C33—C37—O8	−163.6 (4)
C17—C13—C14—C18	−4.2 (8)	C32—C33—C37—O8	8.9 (6)
C12—C13—C14—S2	−0.2 (6)	C34—C33—C37—C40	16.5 (7)
C17—C13—C14—S2	175.9 (4)	C32—C33—C37—C40	−170.9 (4)
C11—C12—C16—O3	−164.6 (4)	C3—C4—S1—C1	−0.4 (4)
C13—C12—C16—O3	9.5 (6)	C8—C4—S1—C1	−178.2 (3)
C11—C12—C16—C19	15.6 (7)	C2—C1—S1—C4	0.2 (4)
C13—C12—C16—C19	−170.2 (4)	C5—C1—S1—C4	−179.4 (4)
C14—C13—C17—O4	61.3 (7)	C12—C11—S2—C14	1.0 (3)
C12—C13—C17—O4	−123.0 (5)	C15—C11—S2—C14	176.8 (3)
C14—C13—C17—C20	−111.7 (5)	C13—C14—S2—C11	−0.4 (4)
C12—C13—C17—C20	63.9 (6)	C18—C14—S2—C11	179.7 (4)
C25—C21—C22—C23	177.8 (4)	C22—C21—S3—C24	1.4 (4)
S3—C21—C22—C23	−0.5 (5)	C25—C21—S3—C24	−177.1 (3)
C25—C21—C22—C26	5.0 (8)	C23—C24—S3—C21	−2.0 (4)
S3—C21—C22—C26	−173.2 (4)	C28—C24—S3—C21	−179.1 (4)
C21—C22—C23—C24	−1.1 (6)	C32—C31—S4—C34	1.7 (4)
C26—C22—C23—C24	172.2 (4)	C35—C31—S4—C34	179.3 (4)
C21—C22—C23—C27	176.3 (4)	C33—C34—S4—C31	−0.9 (3)
C26—C22—C23—C27	−10.4 (7)	C38—C34—S4—C31	176.6 (3)

### Hydrogen-bond geometry (Å, °)

**Table:**

*D*—H···*A*	*D*—H	H···*A*	*D*···*A*	*D*—H···*A*
C30—H30B···O5	0.96	2.48	2.978 (4)	112
C35—H35C···O6^i^	0.96	2.44	3.276 (6)	145
C39—H39A···O6^i^	0.96	2.56	3.435 (6)	152

Symmetry codes: (i) *x*−1, *y*, *z*.
